# The Effect of Artificial Mowing on the Competition of* Phragmites australis* and* Spartina alterniflora* in the Yangtze Estuary

**DOI:** 10.1155/2017/7853491

**Published:** 2017-02-28

**Authors:** Yue Yuan, Chao Zhang, Dezhi Li

**Affiliations:** ^1^Research Institute of Forestry Policy and Information, Chinese Academy of Forestry, Beijing 100091, China; ^2^Key Laboratory of Geographic Information Science, Ministry of Education, East China Normal University, Shanghai 200241, China; ^3^State Key Laboratory of Estuarine and Coastal Research, East China Normal University, Shanghai 200062, China; ^4^Department of Environmental Science, East China Normal University, 500 N. Dongchuan Rd, Shanghai 200241, China; ^5^Shanghai Key Laboratory of Urbanization and Ecological Restoration, Shanghai, China; ^6^National Field Observation and Research Station in the Tiantong Forest Ecosystem of Zhejiang, Ningbo, China

## Abstract

*Spartina alterniflora* Loisel. is one of the most invasive species in the world. However, little is known about the role of artificial mowing in its invasiveness and competiveness. In this work, we studied the effect of mowing on its interspecific interactions with native species* Phragmites australis* (Cav.) Trin ex Steud of the Yangtze Estuary, China. We calculated their relative neighbor effect (RNE) index, effect of relative crowding (*D*_*r*_) index, and interaction strength (*I*) index. The results showed that the RNE of* Phragmites australis* and* Spartina alterniflora* was 0.354 and 0.619, respectively, and they have competitive interactions. The mowing treatments can significantly influence the RNE of* Phragmites australis* and* Spartina alterniflora* on each other. Concretely, the RNE of* Spartina alterniflora* in the removal treatments was significantly higher than the value in the controls. But the RNE of* Phragmites australis* in the removal treatments was significantly lower than the value in the controls. Meanwhile, *D*_*r*_ of the two species on the targets was higher in the removal treatments than that in the controls, and the opposite was for *I*. We concluded that artificial mowing could promote the invasion of* Spartina alterniflora* by increasing its competitive performance compared with native species.

## 1. Introduction

The problem of invasive species and their control has become one of the most pressing applied issues in ecology today [1]. More and more attention was paid to invasive species and the research on the interactions between invasive and native species was thought to be important for biological control [2]. Plant-plant interactions are key processes that strongly influence the composition and structure of plant communities, and both biotic and abiotic factors can influence the outcome of interspecific interactions [3]. Artificial mowing can influence the interactions between invasive species and native species through increasing the availability of both light and nutrients and further to affect species richness, composition, and the competition of species [4, 5].

Salt marsh is susceptible to biological invasion and the invasion has dramatic consequences that include local extinctions of native species, genetic modifications, species displacements, and habitat degradation [6].* Spartina alterniflora* Loisel. (smooth cordgrass) was widely recognized as one of the most aggressive invaders of estuaries and salt marshes around the world [7]. A large number of studies have been carried out on its growth and reproduction and control managements [8, 9]. The ecological measures of controlling* Spartina alterniflora* expansion include chemical herbicide, physical measures, and biological control [10].

The well-known invasive* Spartina alterniflora* was intentionally introduced to China in 2001 for the purpose of land reclamation and the prevention of soil erosion [11, 12]. Its introduction has caused severe ecological consequences to the native ecosystems including conversion of mudflats to* Spartina* meadows, decrease in abundance of native species, considerable loss of shorebirds' foraging habitats, and degradation of native ecosystems. Dongtan wetland of Chongming, East China, which is a typical tidal marsh with an environmental gradient, has also introduced the species in the 1970s and 1980s [13]. The introduction and spread of the alien species resulted in a decrease in the bird biodiversity and abundance of some native species such as* Scirpus triqueter* [11, 14, 15].* Phragmites australis* (Cav.) Trin. ex Steud (common reed), a native species in the Dongtan wetland, coexisted with invasive* Spartina alterniflora*, and they have very similar competitive abilities [16]. Therefore, the biological control of* Spartina alterniflora* by* Phragmites australis* has been a focus of increasing management concern of the government and the ecological scholars.

Some studies have compared the competitive abilities of* Phragmites australis* and* Spartina alterniflora* [17, 18]. And we have known that the invasion of* Spartina alterniflora* and its competition with native species is dramatically influenced by abiotic factors such as salt and water [19]. It seems that no consistent conclusion on their interactions of the two species has been reached. Most of them indicated that the relative competitive ability of* Spartina alterniflora* was significantly greater than that of* Phragmites australis*, but some studies indicated that their interactions may be competitive or facilitative owing to different study conditions [15, 20–22]. We thought the plant interactions of the two species in our research site were competitive according to our early works [16]. However, the role of artificial mowing in their interspecific interactions has not yet been clear.

Herbivores and artificial mowing are very common in the Dongtan wetland of Chongming, East China, and these activities influenced the growth and breed of the species in the area. Mowing could respond in a density-dependent fashion and make plants less able to compete with either conspecifics or surrounding vegetation [5]. Therefore, artificial mowing may influence the control effects of* Spartina alterniflora* by* Phragmites australis*. In this study, we conducted a mowing experiment to study the effect of artificial mowing on the interspecific interactions of* Phragmites australis* and* Spartina alterniflora*.

We evaluated the effects of artificial mowing on the interspecific interactions of* Phragmites australis* and* Spartina alterniflora* by comparing their competitive performance and competitive intensities in different artificial mowing intensity. To evaluate competitive intensities, we chose three measuring indices: relative neighbor effect (RNE), relative crowding (*D*_*r*_), and interaction strength (*I*). We attempted to address the following questions: what is the interspecific interaction of* Phragmites australis* and* Spartina alterniflora* in the research site? How can artificial mowing influence their interactions?

## 2. Materials and Methods

### 2.1. Study Site

**Figure 1:**
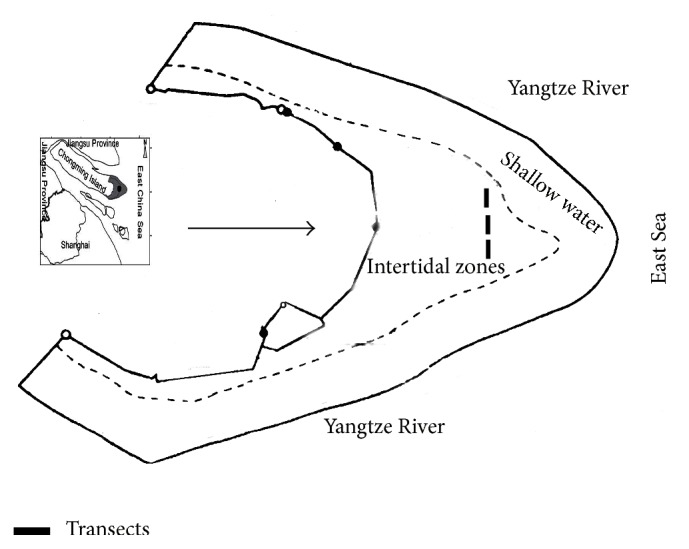
The transects setting in the middle-low tidal zone of Chongming Dongtan wetland, China.

The field studies were conducted at the 32,600 ha Dongtan wetland (31°25′–31°38′N, 121°50′–122°05′E), which is located at the eastern end of Chongming island in the Yangtze River estuary [23]. Dongtan wetland includes both natural and artificial wetlands. The natural wetlands include the intertidal zones and the coastal shallow-water zones below the mean-low-water lines (Figure 1) [19]. The Dongtan wetland was very productive and affected by the semidiurnal tides. Due to the repeated flooding, the intertidal zones were divided into high tidal zone, middle tidal zone, and low tidal zone. The wetlands were 8 km wide at its maximum width in the intertidal zones, with the uppermost 2.5 km covered by marsh vegetation [16].

**Figure 2:**
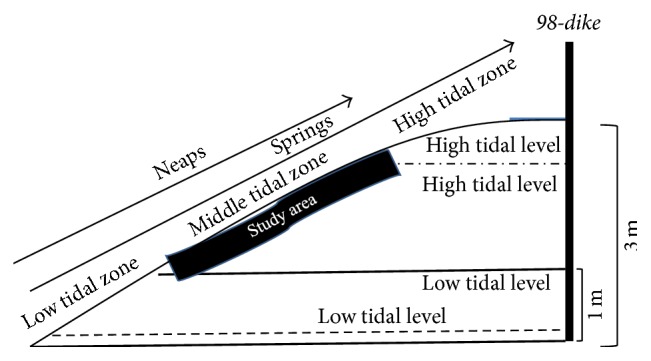
A sketch map on the tidal range and different tidal zones of Chongming Dongtan wetland, China.

The average tidal range of Dongtan wetland was between 2.4 and 3.0 m. The high tidal zone was located between the high tidal level of springs and neaps and its altitude was above 2.5 m. The middle tidal zone was located between the high tidal level of neaps and the low tidal level of neaps and its altitude was between 2.5 m and 1 m. The low tidal zone was located between the low tidal level of springs and neaps and its altitude was below 1 m (Figure 2) [24].

With an elevation increase in the intertidal zones, the water content and the content of NaCl in the soil were significantly different. In the high tidal zone, the water content was approximately 34% to 35%, and the NaCl content was approximately 14 to 25 ppt. In the middle tidal zone, the water content was approximately 27%–32%, and the NaCl content was approximately 25 to 34 ppt. In the low tidal zone, the water content was approximately 33% to 39%, and the NaCl content was approximately 11 to 21 ppt [17].

Before the invasion, the plant communities of Dongtan wetland showed distinct zonation patterns, which were dominated by* Phragmites australis* in the high tidal zone and* Scirpus triqueter* in the low tidal zone [25]. After the invasion,* Scirpus triqueter* in the low tidal zone was completely replaced by* Spartina alterniflora* [26], and* Spartina alterniflora* and* Phragmites australis* coexisted in the wide range of intertidal zones.

### 2.2. Mowing Treatments

We eliminated the aboveground parts of* Spartina alterniflora* and* Phragmites australis* from the plots by a sharp scissors for the artificial mowing treatments. Two intensities were set up: total-removal (TR) in which all of the individuals in plots were eliminated and half-removal (HR) in which half density of the individuals in plots was eliminated.

In July 2010, one single* Phragmites australis* transect, one single* Spartina alterniflora* transect, and one* Phragmites-Spartina* mixtures transect in the middle-low tidal zone of the Dongtan wetlands were set subjectively along the same horizontal line perpendicular to the flooding gradient. All of the three transects were 10 m in length and 3 m in width. Then the three transects were all divided into 30 plots and each plot was 1 m × 1 m. Among that, 10 plots in each transects were chosen randomly as control plots in which no plant individuals were eliminated, 10 plots in each transects were chosen randomly as HR plots, and 10 plots in each transects were chosen randomly as TR plots.

### 2.3. Competitive Intensity

We calculated the competitive intensity for the two species by comparing the performance of the plant in the single species plots and mixture species plots. We chose three measuring indices: relative neighbor effect (RNE), relative crowding (*D*_*r*_), and interaction strength (*I*).

RNE is one of the most widely used indices for measuring the outcomes of competition on organisms [21, 27], which compares the performance of plants growing in neighbor-absence with neighbor-presence conditions. However, increased neighbor crowding may influence the performance of targets and the traditional RNE index does not consider the influence of different neighbor densities on the competitive intensity. Therefore, the RNE cannot distinguish between pure crowding and the actual strength of plant-plant interactions. Considering this limitation, we further calculated *D*_*r*_ and *I* of the two species, which were proposed by Wilson, to distinguish between the influence of pure crowding and the actual strength of plant-plant interactions on RNE. The RNE is equal to the product of the *D*_*r*_ by *I*. For a detailed modeling process, refer to [28].

RNE is calculated as follows:

(1) $$\begin{array}{c} RNE=\frac{\left(y_{iso}-y_{mix}\right)}{\max\left|y_{iso}\right|\text{or}\left|y_{mix}\right|}, \end{array}$$

where *y*_iso_ is the performance of the target species in the single species plots. *y*_mix_ is the performance of the target species in the mixture species plots [27]. In our experiment, the performance of the target species was defined as the relative growth rate (RGR) of the highest individuals per day per plot.

*D*_*r*_ and *I* are calculated as follows:

(2) Dr=zmixmax⁡yiso,ymixI=yiso−ymixzmix,

where *z*_mix_ is the abundance of neighbors surrounding the target plants. *y*_iso_ is the performance of a target plant grown in the single species plots, and *y*_mix_ is the performance of a target plant grown in the mixture species plots [28]. Similarly, the performance of the target species was defined as the RGR of the highest individuals per day per plot.

The RGR of the highest individuals per day per plot was calculated using

(3) $$\begin{array}{c} RGR=\frac{\left[\ln\left(M_{2}\right)-\ln\left(M_{1}\right)\right]}{\left(t_{2}-t_{1}\right)}, \end{array}$$

where *M*_2_ and *M*_1_, respectively, represent the aboveground biomass of the highest individuals in plots before and after the treatments and *t*_2_ − *t*_1_ is the number of days of the experiment. Here our experiment lasted for 120 days.

To calculate the RGR, we needed to know the target biomass both before and after the treatments. Therefore, six higher individuals per plot were selected as the highest individuals. They were marked and distributed randomly and evenly for the measurement of the target biomass both before and after the treatments. Therefore, the target biomass before and after the treatments here was the average value of the three higher individuals biomass. We mowed the aboveground parts of the targets and oven-dried them to a constant weight at 70°C to estimate the target biomass.

### 2.4. Statistics

Through above calculations, we compared the mean competitive intensities of* Phragmites australis* and* Spartina alterniflora* among different mowing treatments. Meanwhile, we also compared the mean height of highest individuals and mean number of* Phragmites australis* and* Spartina alterniflora* individuals per plot among different mowing treatments.

In our research, all statistical analyses were conducted using SAS 8.1 (SAS Institute Inc., USA). We analyzed the experimental results using an analysis of variance (ANOVA). Normality and homoscedasticity of all data were tested first. Data that violated these assumptions were ln-transformed to improve normality and homoscedasticity. The statistical tests were considered significant at the 0.05 probability level.

## 3. Results

### 3.1. The Effect of Different Mowing Intensities on* Phragmites australis *and* Spartina alterniflora*

#### 3.1.1. The Height of Highest Individuals

**Figure 3:**
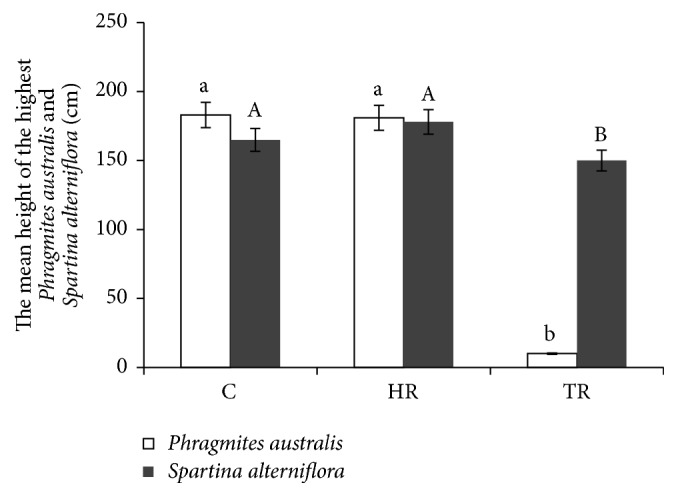
The height of the highest* Phragmites australis* and* Spartina alterniflora* (mean ± SE) in different mowing treatments: control (C), HR (half-removal), and TR (total-removal). Different letters indicate a significant difference (*P* < 0.05) among treatments.

The analysis of variance indicated that TR treatments significantly affected the height of the highest individuals of* Phragmites australis* and* Spartina alterniflora*. At the end of the treatments, the mean height of the highest individuals of* Phragmites australis* and* Spartina alterniflora* in the TR treatments was significantly lower than that in the controls (*P* < 0.05), but the mean height of the highest individuals of* Phragmites australis* and* Spartina alterniflora* in the HR treatments was similar to that in the control treatments (Figure 3). The result showed that only higher mowing intensity could affect the growth performance of* Phragmites australis* and* Spartina alterniflora*.

#### 3.1.2. The Number of Individuals per Plot

**Figure 4:**
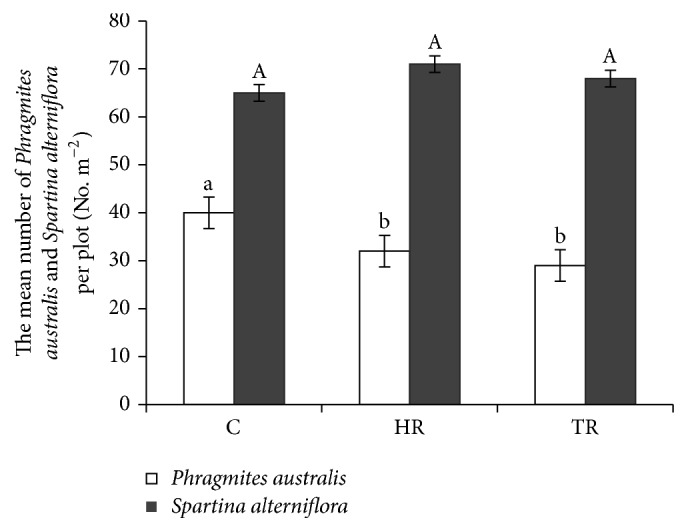
The number of* Phragmites australis* and* Spartina alterniflora* individuals per plot (mean ± SE) in different mowing treatments: control (C), HR (half-removal), and TR (total-removal). Different letters indicate a significant difference (*P* < 0.05) among treatments.

The analysis of variance indicated that mowing treatments significantly affected the number of* Phragmites australis* individuals per plot (*P* < 0.05). In detail, the mean number of* Phragmites australis* individuals per plot was higher in the control treatments than that in the TR and HR treatments at the end of the treatments, but the mean number of* Phragmites australis* individuals per plot in the HR treatments was similar to that in the TR treatments. There were no significant differences in the number of* Spartina alterniflora* individuals per plot between the different mowing treatments and the controls (Figure 4).

### 3.2. Competitive Intensities of* Phragmites australis *and* Spartina alterniflora* on Each Other in Different Mowing Treatments

#### 3.2.1. RNE

A negative competitive effect was found in the mixed community for* Phragmites australis* and* Spartina alterniflora*, and the effect of* Spartina alterniflora* on* Phragmites australis* was even more pronounced. The mean RNE of* Phragmites australis* and* Spartina alterniflora* on each other in the controls was 0.354 and 0.619, respectively.

The relationship between RNE and mowing treatments was not the same for* Spartina alterniflora* and* Phragmites australis*. The mean RNE of* Spartina alterniflora* on the targets in the HR and TR treatments was 0.963 and 1.000, respectively, and expected to be more intense than that in the controls. However, the mean RNE of* Phragmites australis* on the targets in the HR and TR treatments was 0.116 and 0.102, respectively, and expected to be more alleviative than that in the controls (Table 1).

#### 3.2.2. *D*_*r*_

The results showed that *D*_*r*_ of* Spartina alterniflora* on* Phragmites australis* was more pronounced than that of* Phragmites australis* on* Spartina alterniflora* in the controls. The mean *D*_*r*_ of* Phragmites australis* and* Spartina alterniflora* on each other in the controls was 1220 and 6881, respectively. Moreover, the relationship between *D*_*r*_ and mowing treatments was the same for* Spartina alterniflora* and* Phragmites australis*. That is, *D*_*r*_ of both* Spartina alterniflora* and* Phragmites australis* on the targets was expected to be more intense when there was a mowing disturbance.

#### 3.2.3. *I*

The results showed that *I* of* Phragmites australis* on* Spartina alterniflora* was more pronounced than that of* Spartina alterniflora* on* Phragmites australis* in the controls. The mean *I* of* Phragmites australis* and* Spartina alterniflora* was 0.00029 and 0.00009, respectively, in the controls. Moreover, the relationship between *I* and mowing treatments was the same for* Spartina alterniflora* and* Phragmites australis*. That is, *I* of both* Spartina alterniflora* and* Phragmites australis* on the targets was expected to be more intense when there was no mowing disturbance.

## 4. Discussions

In this study we investigated the response of plant-plant interactions between invasive and native species towards artificial mowing in a typical salt marsh of China. Our results found that artificial mowing had a positive influence on the competitive effect of* Spartina alterniflora* on* Phragmites australis* and* a* negative influence on the competitive effect of* Phragmites australis* on* Spartina alterniflora.*

### 4.1. Affecting Factors of Plant Competitive Abilities

One species may have specific traits that allow them to outcompete other species such as fast growth, rapid reproduction, high dispersal ability, phenotypic plasticity, and association with humans [29]. For* Spartina alterniflora* and* Phragmites* australis, difference of photosynthesis type may result in different plant growth abilities of the two species.* Phragmites australis* is a C_3_ plant, which has the obvious photosynthesis midday depression phenomenon. While* Spartina alterniflora* is a C_4_ plant with higher apparent quanta efficiency, carboxylic efficiency, and net photosynthetic rate [22]. In addition,* Phragmites australis* and* Spartina alterniflora* have different absorptive capacity for nitrogen [30, 31]. With the increase of the content of soil nitrogen,* Phragmites australis* is growing better. Therefore, the relative performance and competitive ability of plants also depend on their growing conditions [17].

Competitive advantage species may have strong genetic differentiation and phenotypic plasticity. Some researchers reported genetic diversity of* Spartina alterniflora* was very high and its Shannon genetic diversity index can reach 0.703. Among that, its intraspecific genetic diversity index was greater than that of interspecific genetic diversity, but there were also differentiation among populations [32]. In addition, some studies indicated that the phenotypic plasticity indices of* Spartina alterniflora* were higher than that of* Phragmites australis* for traits related to the morphology, growth, and biomass allocation in response to nitrogen and culm density [33, 34].

### 4.2. The Influence of Mowing on the Interspecific Interactions between Invasive and Native Species

In the researches of invasive mechanism, both species and ecosystem factors should be considered. Many hypotheses have been proposed to explain the success of plant invasions. For example, a common explanation is that invasive species outcompete their cooccurring natives [15, 35, 36]. However, some studies also found invasive species tended to have many similar traits compared with native species in some physical conditions [37].

Disturbance may have an influence on the competitive abilities of different species. Shifts in the relative availability of canopy resources versus soil resources might modulate interspecific competition and, therefore, the outcome of mowing on community structure [38]. Tissue loss and modified light profiles may be major causes of changes in establishment, growth, competitive success, and longevity [39]. Mowing hardly causes the instantaneous death of individuals but rather removes aboveground biomass and often acts selectively towards certain plant trait attributes, such as large height and palatable leaf tissue. Mowing is also supposed to change the relative importance of aboveground versus below-ground competition and thus potentially the competitive hierarchy among species [40]. Humans promoted or inhibited invaders through exerting their influence on the interspecific interactions of native and invasive species.

Disturbance has a long and recurring role as a potential explanation for the coexistence of species and the maintenance of patterns of species diversity. The human release hypothesis stated that the abundance of invasive species is different between different regions because population expansion is reduced in some regions through continuous land management and associated cutting of the invasive species [41–46]. Invaded ecosystems may have experienced disturbance, typically human-induced. Such a disturbance may give invasive species a chance to establish themselves with less competition from natives less able to adapt to a disturbed ecosystem.

### 4.3. Control Suggestions for Invasive Species

Concretely, some studies indicated controlled water-logging was an effective measure to the invasive plant* Spartina alterniflora* [47], and some studies indicated clipping vegetation at the early florescence stage and the integrated technique of cutting plus water-logging were more efficient for controlling the invasive plant* Spartina alterniflora* [48]. Moreover, 3S (GPS, RS, and GIS) technology, mathematical models of population growth and dispersal, and long-term monitoring systems have also been employed in* Spartina alterniflora* monitoring and controlling [49].

*Spartina alterniflora* may invade new places by quickly occupying more space and inhibiting the growth of* Phragmites australis*. Meanwhile,* Phragmites australis* might have a genetic competitive dominance over* Spartina alterniflora* because of its strong *I*. Therefore, the control of* Spartina alterniflora* by* Phragmites australis* is feasible. In addition, the competitive intensity of* Spartina alterniflora* would be increased, and the competitive intensity of* Phragmites australis* would be decreased if there was a disturbance. Artificial mowing disturbance may promote the spread of* Spartina alterniflora* in the Dongtan wetland. Meanwhile, grazing and human mowing are very common in this area, and these activities have important influences on the growth and colonize of* Spartina alterniflora* [50]. Therefore, we suggested that wetland managers should plan to establish nature reserves and prohibit the harvesting behavior of local residents for the better control of invasive* Spartina alterniflora*.

## 5. Conclusions

Our results found that* Spartina alterniflora* has a competitive dominance over* Phragmites australis* in the middle-low tidal zone of Dongtan wetland on the eastern coast of China. Our results also showed that RNE of* Spartina alterniflora* on* Phragmites australis* increased with human mowing intensities, but the RNE of* Phragmites australis* on* Spartina alterniflora* decreased with human mowing intensities.

In our study, *D*_*r*_ was higher for both* Spartina alterniflora* and* Phragmites australis* in the mowing treatments than that in the controls, but *I* was lower for both* Spartina alterniflora* and* Phragmites australis* in the mowing treatments than that in the controls. The change of RNE of* Spartina alterniflora* with the mowing treatments was the same as the change of *D*_*r*_ of* Spartina alterniflora* with the mowing treatments. And the change of RNE of* Phragmites australis* with the mowing treatments was the same as the change of *I* of* Phragmites australis* with the mowing treatments. RNE of* Spartina alterniflora* may be mainly determined by its *D*_*r*_, and RNE of* Phragmites australis* may be mainly determined by its *I*.

## Figures and Tables

**Table 1:** The RNE, *D*_*r*_, and *I* of *Phragmites australis* and *Spartina alterniflora *in different mowing treatments (*n* = 10 plots) (mean ± SE).

	RNE	*D*_*r*_	*I*
*Spartina alterniflora*			
Control	0.619 ± 0.018	6881 ± 239.145	0.00009 ± 0.000
HR	0.963 ± 0.041^*∗*^	12400 ± 998.395^*∗∗*^	0.00007 ± 0.000^*∗∗*^
TR	1.000 ± 0.000^*∗∗*^	15926 ± 1203.326^*∗∗*^	0.00006 ± 0.000^*∗∗*^

*Phragmites australis*			
Control	0.354 ± 0.046	1220 ± 101.444	0.00029 ± 0.000
HR	0.116 ± 0.039^*∗*^	2318 ± 265.353^*∗∗*^	0.00005 ± 0.0000^*∗∗*^
TR	0.102 ± 0.026^*∗∗*^	2547 ± 278.355^*∗∗*^	0.00004 ± 0.000^*∗∗*^

HR means half-removal treatments. TR means total-removal treatments.

^*∗*^ *P* < 0.05; ^*∗∗*^*P* < 0.01.
